# Cross-Sectional and Longitudinal Replication Analyses of Genome-Wide Association Loci of Type 2 Diabetes in Han Chinese

**DOI:** 10.1371/journal.pone.0091790

**Published:** 2014-03-17

**Authors:** Qi Zhao, Jianzhong Xiao, Jiang He, Xuelian Zhang, Jing Hong, Xiaomu Kong, Katherine T. Mills, Jianping Weng, Weiping Jia, Wenying Yang

**Affiliations:** 1 Department of Epidemiology, Tulane University School of Public Health and Tropical Medicine, New Orleans, Louisiana, United States of America; 2 Department of Endocrinology, China-Japan Friendship Hospital, Beijing, China; 3 Department of Medicine, Tulane University School of Medicine, New Orleans, Louisiana, United States of America; 4 Sun Yat-Sen University Third Affiliated Hospital, Guangzhou, Guangdong, China; 5 Shanghai Jiaotong University Affiliated Sixth People's Hospital, Shanghai, China; Sanjay Gandhi Medical Institute, India

## Abstract

This study aimed to examine genomic loci of type 2 diabetes (T2D) initially identified by genome-wide association studies in populations of European ancestry for their associations with T2D and quantitative glycemic traits, as well as their effects on longitudinal change in fasting plasma glucose (FPG) and T2D development, in the Chinese population. Single nucleotide polymorphisms (SNP) from 25 loci were genotyped in a large case-control sample of 10,001 subjects (5,338 T2D cases and 4,663 controls) and a prospective cohort of 1,881 Chinese. In the case-control sample, 8 SNPs in or near *WFS1*, *CDKAL1*, *CDKN2A/2B*, *CDC123*, *HHEX*, *TCF7L2*, *KCNQ1*, and *MTNR1B* were significantly associated with T2D (*P*<0.05). Thirteen SNPs were associated with quantitative glycemic traits. For example, the most significant SNP, rs10811661 near *CDKN2A*/*2B* (*P* = 1.11×10^−8^ for T2D), was also associated with 2-h glucose level of an oral glucose tolerance test (*P* = 9.11×10^−3^) and insulinogenic index (*P* = 2.71×10^−2^). In the cohort study, individuals carrying more risk alleles of the replicated SNPs had greater FPG increase and T2D incidence in a 7.5-year follow-up period, with each quartile increase in the number of risk alleles being associated with a 0.06 mmol/l greater increase in FPG (*P* = 0.03) and 19% higher odds of developing T2D (*P* = 0.058). Our study identified the associations of several established T2D-loci in Europeans with T2D and quantitative glycemic traits in the Chinese population. The prospective data also suggest their potential role in the risk prediction of T2D in the Chinese population.

## Introduction

China has experienced an explosive increase in the prevalence of diabetes in the past two decades [1]. Although environmental and lifestyle factors undoubtedly contribute to the increase of type 2 diabetes (T2D) in China, genetic factors determine individual susceptibility to these risk factors. Multiple lines of evidence have indicated that T2D and its related glycemic traits have considerable heritability [2], [3]. Recent genome-wide association studies (GWAS) identified more than 60 novel genomic loci associated with T2D, which greatly advanced the understanding of the genetic basis of T2D [4].

Because of the potential genetic heterogeneity of T2D across populations of different racial/ethnic backgrounds and because most novel GWAS-loci were initially identified in populations of European ancestry, it is necessary to replicate the association of these novel loci with T2D in independent populations with various ethnicities. Several genetic replication studies have been conducted in Chinese populations and reported inconsistent findings on these associations [5]–[8]. There is still lack of evidence for the associations between the T2D-related loci identified in populations of European ancestry and T2D in Han Chinese, which constitutes the majority of Chinese population. In addition, the associations of these novel loci with 2-h postload glucose level after an oral glucose tolerance test (OGTT) and insulin resistance measures have not been examined among the Chinese population.

In the current study, we tested the association of established T2D-loci in populations of European ancestry and T2D among a large case-control sample of Han Chinese and investigated their associations with quantitative glycemic traits. In addition, we studied the cumulative effects of significant loci on changes in fasting glucose and the incidence of T2D among study participants from north rural China of the Genetic Epidemiology Network of Salt Sensitivity (GenSalt) over more than 7 years of follow-up.

## Methods

### Study subjects

#### The DMS case-control sample

The China National Diabetes and Metabolic Disorders Study (DMS) collected a nationally representative sample of 46,239 adults aged 20 years or older from 14 provinces and municipalities in China [9]. After at least 10 hours of overnight fasting, a venous blood specimen was collected. Then, all study participants underwent a 75-g oral glucose-tolerance test (OGTT). The fasting and 2-h glucose levels were measured to identify undiagnosed diabetes (fasting plasma glucose (FPG) ≥ 7.0 mmol/l and/or 2-h OGTT glucose ≥11.1 mmol/l), while previously diagnosed diabetes was determined by self-report. A total of 5,338 participants with T2D were identified and included as cases in this study. A random sample of 4,663 healthy participants without T2D or pre-diabetes (FPG<6.1 mmol/l and 2-h OGTT glucose<7.8 mmol/l) was included as controls.

#### The GenSalt cohort study sample

The GenSalt study is a family-based dietary feeding study designed to investigate genetic factors associated with BP response to dietary sodium and potassium interventions among a Han Chinese population [10]. A community-based BP screening was conducted among people 18–60 years of age who resided in the study villages to identify potential probands and their families. Probands with prehypertension or stage-1 hypertension and no use of antihypertensive medications were recruited for dietary interventions, along with their siblings, spouses, and offspring. The participant recruitment and baseline data collection were conducted from 2003 to 2005. Two follow-up examinations were completed in 2008 and 2011, respectively. A total of 1,881 GenSalt study participants without T2D at baseline were included in the current study. Data on T2D diagnosis and treatment and FPG was collected at baseline and follow-up visits.

The DMS and GenSalt studies were approved by the Ethics Committee of China-Japan Friendship Hospital and the Institutional Review Board of Tulane University, respectively. Written informed consent was obtained from each participant of the two studies.

### Quantitative glycemic trait measurements

In the DMS study, blood samples were obtained at baseline after fasting and at 30 minutes and 2 hours after oral glucose administration during the OGTT in all study participants. Plasma glucose was measured with the use of a hexokinase enzymatic method and serum insulin was measured by double-antibody radioimmunoassay. Indices of beta-cell function (HOMA-B) and insulin resistance (HOMA-IR) were derived from paired fasting glucose and insulin measures using homeostasis model assessment [11]. In addition, insulinogenic index was calculated using the formula (insulin at 30 minutes – insulin at 0 minutes)/(glucose at 30 minutes – glucose at 0 minutes) to assess the early insulin secretion phase in response to the oral glucose challenge [12]. In the GenSalt study, FPG was measured during baseline and follow-up examinations using the hexokinase enzymatic method.

### SNP selection and genotyping

A total of 29 single nucleotide polymorphisms (SNPs) from 28 established T2D loci in populations of European ancestry were genotyped among the DMS case-control sample using the Illumina GoldenGate Indexing assay (Illumina Inc., San Diego, CA). Twenty-five SNPs from 25 loci were successfully genotyped with an average call rate of 98.4% (Table S1 in File S1). Two of these SNPs, rs1801282 and rs7578597, are non-synonymous SNPs and are predicted to have potential impacts on exonic splicing. There are no predicted functions for the other SNPs based on the SNPinfo database (http://snpinfo.niehs.nih.gov/snpinfo/snpfunc.htm), a web tool for SNP function prediction [13]. The concordance rate was 100% for 229 duplicate samples. The genotypes of the selected SNPs were extracted from the genotyped (Affymetrix Genomewide Human SNP array 6.0 (Affymetrix, Inc., Santa Clara, CA)) and imputed data of the GenSalt sample [14].

### Statistical analyses

Each SNP was tested for deviation from the Hardy-Weinberg equilibrium (HWE) within the DMS control group and the GenSalt sample using an exact test implemented in Haploview software [15]. In the DMS case-control sample, an additive genetic model with age and sex as covariates was used to test for the association of each SNP with T2D using logistic regression models. Body mass index (BMI) was further adjusted for in these models to examine whether a SNP's effect on T2D was independent of BMI. Associations between SNPs and quantitative glycemic traits under an additive genetic model were analyzed among DMS controls using general linear models that included age and sex. BMI was further adjusted in these models. Log-transformed values for fasting insulin, HOMA-B, HOMA-IR, and insulinogenic index were used as dependent variables.

During the conduction of this study, the association results of 22 genotyped SNPs in this study became available in the Asian Genetic Epidemiology Network (AGEN) consortium, which included 6,952 T2D cases and 11,865 controls of East Asian descent in its GWAS meta-analysis discovery stage. To provide more precise estimates of effect sizes for risk alleles of the tested SNPs in East Asians, we conducted a meta-analysis to combine our results with those from the Asian Genetic Epidemiology Network (AGEN) consortium using a fixed effects model weighted by inverse variance [14].

To test the effect of the replicated SNPs on long-term change in FPG and the development of T2D, a genetic risk score was calculated for each individual in the GenSalt sample. The sum of the number of risk alleles at each SNP was weighted according to the SNP's relative effect size which was derived from the meta-analysis of AGEN and this study. We rescaled the weighted score to reflect the number of risk alleles each individual carried, and each point of the genetic-predisposition score corresponded to one risk allele [16]. Generalized estimating equations were used to test the associations of genetic risk score with FPG change and T2D incidence over follow-up accounting for non-independence of GenSalt family members. Age, sex, and baseline BMI were adjusted in these models. SAS statistical software (version 9.2; SAS Institute Inc., Cary, NC) was used to conduct association analyses.

## Results

The clinical characteristics of the DMS case-control sample and the baseline characteristics of GenSalt participants are shown in Table 1. None of the SNPs deviated significantly from HWE after correcting for multiple testing among the control sample of the DMS study (the smallest *P*-value  = 0.02, Table S1 in File S1). In addition, allele frequencies of the genotyped SNPs in this study were close to those observed among Han Chinese individuals from Beijing (CHB) in the HapMap project (Table S1 in File S1).

**Table 1:** Characteristics of study participants.

	DMS		GenSalt
	Case (N = 5,338)	Control (N = 4,663)	N = 1,881
Age, year	55.0 (11.8)	50.7 (8.4)	38.7 (9.5)
Male, %	43.4	32.2	52.8
Body mass index, kg/m^2^	25.9 (3.7)	23.0 (2.5)	23.3 (3.2)
Waist circumference, cm	88.2 (10.0)	79.0 (8.5)	80.3 (9.8)
Fasting glucose, mmol/l	8.0 (2.7)	5.0 (0.5)	4.8 (0.7)
2-Hour glucose in OGTT, mmol/l	14.2 (5.2)	5.7 (1.1)	-
Fasting insulin, pmol/l	60.7 (42.2–87.5)	43.7 (33.8–58.7)	-
HOMA-B, %	46.9 (27.9–77.0)	85.3 (60.6–125.3)	-
HOMA-IR	3.0 (1.9–4.6)	1.4 (1.1–1.9)	-
Insulinogenic index	2.3 (0.7–5.5)	8.9 (4.5–16.7)	-
Diabetes Treatment, %	37.5	0	0

Continuous variables are presented as mean (standard deviation) or median (interquartile range). DMS, the China National Diabetes and Metabolic Disorders study; GenSalt, the Genetic Epidemiology Network of Salt Sensitivity; HOMA-B, homoeostasis model assessment of beta-cell function; HOMA-IR, homoeostasis model assessment of insulin resistance; OGTT, oral glucose tolerance test.

### Association analyses with T2D in the DMS study

In the DMS sample, 9 SNPs were significantly associated with T2D (*P*<0.05) in logistic regression analysis without adjustment for BMI (Table S2 in File S1). The associations of the SNP of the *FTO* gene (rs9939609) were no longer significant after adjusting for BMI, leaving 8 SNPs significantly associated with T2D (Table 2). Five loci including SNPs within or near *CDKAL1*, *CDKN2A/2B*, *HHEX*, *TCF7L2* and *KCNQ1* remained significant even after correcting for multiple testing using the Bonferroni method (*P*<0.05/26 = 0.002, Table 2). Among these loci, SNP rs10811661 of the *CDKN2A/2B* reached genome-wide significance (*P* = 1.11×10^−8^).

**Table 2:** Association results for replicated loci (*P*<0.05) in DMS and combined DMS+AGEN analyses.

							DMS^b^		DMS + AGEN		
SNP	Chr	Physical Position	Nearby gene	Gene Region	Alleles^a^	MAF	OR (95% CI)	*P*-value	OR (95% CI)	*P*-value	Reported OR^c^
rs780094	2	27741237	*GCKR*	Intronic	A:**G**	0.476	1.06 (0.99–1.13)	0.08	1.05 (1.02–1.09)	**4.52×10^−3^**	1.06
rs243021	2	60584819	*BCL11A* d	Intergenic	**T**:C	0.320	1.02 (0.96–1.10)	0.49	1.04 (1.00–1.08)	**0.04**	1.08
rs1801282	3	12393125	*PPARG*	Exonic	**C**:G	0.065	1.10 (0.96–1.25)	0.17	1.12 (1.02–1.22)	**0.02**	1.14
rs10010131	4	6292915	*WFS1*	Intronic	**G**:A	0.046	1.21 (1.04–1.41)	**0.02**	1.05 (0.97–1.14)	0.21	1.11
rs7756992	6	20679709	*CDKAL1*	Intronic	**G**:A	0.477	1.16 (1.08–1.23)	**1.02×10^−5^**	-	-	1.20
rs864745	7	28180556	*JAZF1*	Intronic	**A**:G	0.239	1.04 (0.96–1.12)	0.37	1.05 (1.00–1.10)	**0.03**	1.10
rs896854	8	95960511	*TP53INP1*	Intronic	G:**A**	0.341	1.02 (0.96–1.10)	0.49	1.06 (1.02–1.10)	**6.30×10^−3^**	1.06
rs10811661	9	22134094	*CDKN2A/B* d	Intergenic	**T**:C	0.476	1.21 (1.13–1.29)	**1.11×10^−8^**	1.21 (1.16–1.26)	**6.87×10^−18^**	1.19
rs12779790	10	12328010	*CDC123* d	Intergenic	A:**G**	0.165	1.14 (1.05–1.24)	**2.27×10^−3^**	1.13 (1.06–1.20)	**1.16×10^−4^**	1.11
rs1111875	10	94462882	*HHEX* d	Intergenic	A:**G**	0.284	1.13 (1.05–1.21)	**8.05×10^−4^**	1.12 (1.07–1,17)	**4.09×10^−7^**	1.13
rs7903146	10	114758349	*TCF7L2*	Intronic	C:**T**	0.039	1.34 (1.15–1.57)	**1.97×10^−4^**	1.23 (1.11–1.36)	**3.41×10^−5^**	1.40
rs2237895	11	2857194	*KCNQ1*	Intronic	A:**C**	0.320	1.22 (1.13–1.31)	**5.45×10^−8^**	-	**-**	1.29
rs1552224	11	72433098	*ARAP1*	Exonic	**T**:G	0.090	1.06 (0.95–1.19)	0.31	1.12 (1.04–1.20)	**1.69×10^−3^**	1.14
rs10830963	11	92708710	*MTNR1B*	Intronic	C:**G**	0.413	1.08 (1.01–1.15)	**0.03**	1.03 (0.99–1.08)	0.18	1.09
rs8042680	15	91521337	*PRC1*	Intronic	**A**:C	0.019	1.12 (0.89–1.42)	0.33	1.27 (1.04–1.54)	**0.02**	1.07
rs9939609	16	53820527	*FTO*	Intronic	T:**A**	0.113	1.09 (0.99–1.21)	0.07	1.13 (1.08–1.19)	**1.91×10^−6^**	1.34

P-values <0.05 are shown in bold in DMS and combined DMS+AGEN analyses.

a Major allele: minor allele; previously reported risk alleles (effect alleles) are shown in bold.

b Association results of the logistic regression analysis with adjustment for body mass index.

c Previously reported effects mainly among Europeans.

d The nearest gene is provided if a SNP is intergenic.

AGEN, the Asian Genetic Epidemiology Network; Chr, chromosome; DMS, the China National Diabetes and Metabolic Disorders study; MAF, minor allele frequency; OR, odds ratio; SNP, single nucleotide polymorphism.

### Meta-analysis of DMS results with published AGEN data

Among the 8 replicated SNPs in the DMS sample, 6 SNPs were available for comparison with the published AGEN results. The associations of 4 SNPs (within or near *CDKN2A/2B*, *CDC123*, *HHEX*, and *TCF7L2*) were further confirmed by the AGEN results (*P*<0.05), showing consistent association directions (Table S2 in File S1). There was no significant heterogeneity for the effects of the 22 SNPs which were available for comparison between the DMS study and the AGEN study (data were not shown). The combined analysis of the DMS and AGEN results showed another 8 significant loci which includes SNPs within or near *GCKR*, *BCL11A*, *PPARG*, *JAZF1*, *TP53INP1*, *ARAP1*, *PRC1*, and *FTO* (*P*<0.05, Table 2). Most of replicated SNPs showed similar effects with those observed in European populations (Table 2).

### Association analyses with quantitative glycemic traits in DMS controls

In the DMS control sample, multiple SNPs showed significant associations with quantitative glycemic traits (*P*<0.05, Table 3 and Table S3 in File S1). The risk allele T of the most significant SNP in the association with T2D, *CDKN2A/2B*-rs10811661, was associated with a higher glucose level during the OGTT (β (SE) = 0.06 (0.02), *P* = 9.11×10^−3^) and a lower insulinogenic index (β (SE) = −0.05 (0.02), *P* = 0.03), suggesting that the role of this locus in T2D may be mediated through β-cell dysfunction. In addition, T2D risk allele G of *CDC123*-rs12779790 was associated with a higher fasting insulin level (β (SE) = 0.03 (0.01), *P* = 8.58×10^−3^) and a greater HOMA-IR (β (SE) = 0.04 (0.01), *P* = 9.39×10^−3^), suggesting that this locus may be involved in insulin resistance.

**Table 3:** Significant associations (*P*<0.05) of reported-T2D loci with quantitative glycemic traits in controls of the DMS case-control sample.

			Fasting glucose		OGTT 2-h glucose		Fasting insulin^b^							
			(mmol)		(mmol/l)		(pmol/l)		HOMA_IR^b^		HOMA_B^b^		Insulinogenic index^b^	
SNP	Nearby gene	Effect allele^a^	β (SE)	*P*-value	β (SE)	*P*-value	β (SE)	*P*-value	β (SE)	*P*-value	β (SE)	*P*-value	β (SE)	*P*-value
rs780094	*GCKR*	G	0.02 (0.01)	0.07	0.03 (0.02)	0.17	0.01 (0.01)	0.43	0.01 (0.01)	0.21	−0.01 (0.01)	0.52	0.05 (0.02)	**0.047**
rs243021	*BCL11A* c	T	0.02 (0.01)	0.055	−0.03 (0.02)	0.24	0.02 (0.01)	0.08	0.02 (0.01)	**0.03**	0 (0.01)	0.91	0.02 (0.03)	0.41
rs4607103	*ADAMTS9* c	C	0.01 (0.01)	0.25	0.02 (0.02)	0.45	0 (0.01)	0.67	0.01 (0.01)	0.56	0 (0.01)	0.95	−0.07 (0.02)	**5.33×10^−3^**
rs7756992	*CDKAL1*	G	0.02 (0.01)	0.09	0.04 (0.02)	0.055	−0.01 (0.01)	0.21	−0.01 (0.01)	0.37	−0.02 (0.01)	0.12	−0.07 (0.02)	**6.21×10^−3^**
rs896854	*TP53INP1*	A	0.01 (0.01)	0.20	−0.01 (0.02)	0.65	−0.03 (0.01)	**0.01**	−0.02 (0.01)	**0.03**	−0.03 (0.01)	**0.04**	−0.03 (0.03)	0.32
rs10811661	*CDKN2A/B* c	T	0.01 (0.01)	0.49	0.06 (0.02)	**9.11×10^−3^**	0 (0.01)	0.96	0 (0.01)	0.78	−0.01 (0.01)	0.53	−0.05 (0.02)	**0.03**
rs13292136	*CHCHD9* c	C	−0.02 (0.02)	0.42	−0.08 (0.04)	**0.03**	−0.02 (0.02)	0.32	−0.02 (0.02)	0.28	−0.01 (0.02)	0.78	−0.04 (0.04)	0.33
rs12779790	*CDC123* c	G	0.01 (0.01)	0.60	0.02 (0.03)	0.42	0.03 (0.01)	**8.58×10^−3^**	0.04 (0.01)	**9.39×10^−3^**	0.02 (0.02)	0.18	0 (0.03)	0.99
rs1111875	*HHEX* c	G	−0.02 (0.01)	0.12	−0.01 (0.03)	0.79	−0.01 (0.01)	0.29	−0.02 (0.01)	0.17	0.01 (0.02)	0.71	−0.09 (0.03)	**6.73×10^−4^**
rs7903146	*TCF7L2*	T	0.03 (0.03)	0.34	0.17 (0.06)	**4.90×10^−3^**	0.03 (0.03)	0.26	0.03 (0.03)	0.24	0.02 (0.04)	0.48	−0.01 (0.06)	0.87
rs10830963	*MTNR1B*	G	0.03 (0.01)	**5.50×10^−3^**	0 (0.02)	0.86	0 (0.01)	0.83	0.01 (0.01)	0.43	−0.02 (0.01)	0.16	−0.02 (0.02)	0.43
rs11634397	*ZFAND6* c	G	−0.03 (0.02)	0.12	−0.05 (0.04)	0.21	0 (0.02)	0.78	0 (0.02)	0.97	0.03 (0.02)	0.15	−0.11 (0.04)	**4.67×10^−3^**
rs9939609	*FTO*	A	−0.01 (0.02)	0.75	0 (0.04)	0.90	−0.04 (0.02)	**0.02**	−0.04 (0.02)	**0.02**	−0.02 (0.02)	0.33	0.01 (0.04)	0.73

*P*-values <0.05 are shown in bold.

a Previously reported risk alleles.

b Log-transformed values were used in general linear regression models.

c The nearest gene is provided if a SNP is intergenic.

HOMA-B, homoeostasis model assessment of beta-cell function; HOMA-IR, homoeostasis model assessment of insulin resistance; OGTT, oral glucose tolerance test; SE, standard error; SNP, single nucleotide polymorphism.

### Cumulative effect of replicated SNPs on the progression to diabetes among GenSalt participants

**Figure 1:**
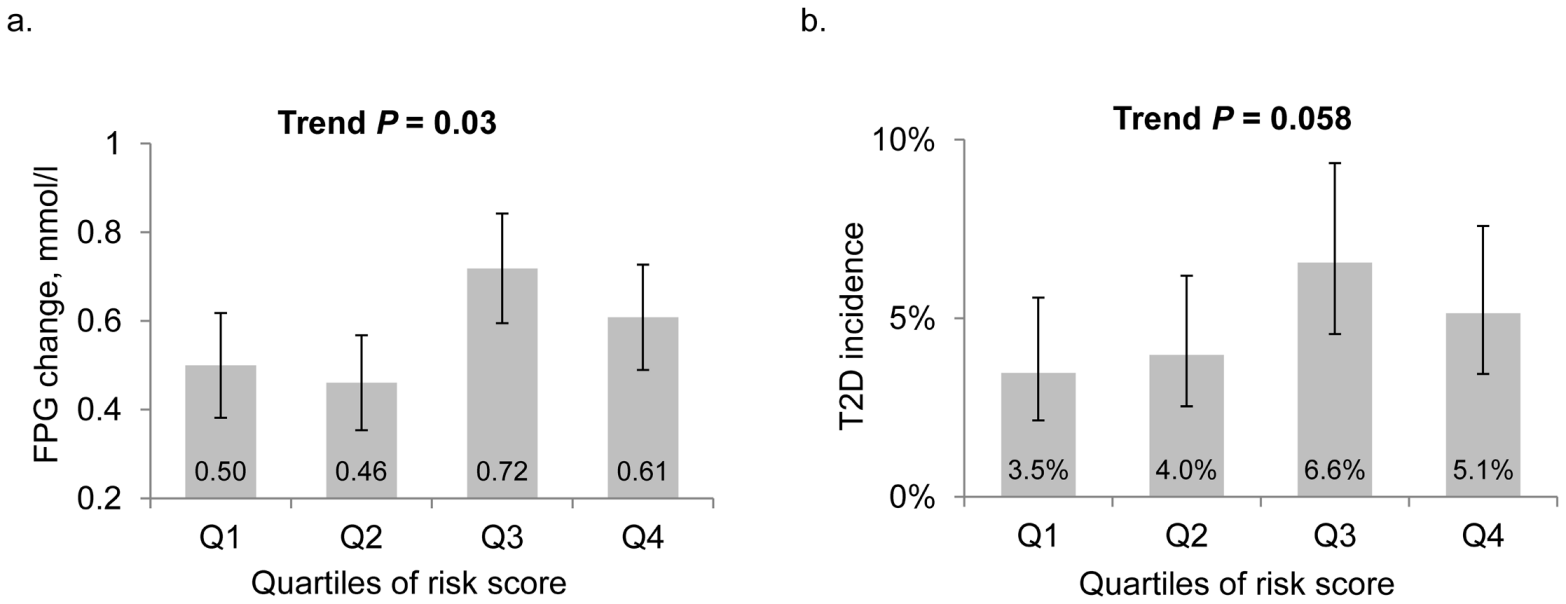
The associations of risk scores with FPG change and accumulative T2D incidence over a 7.5-year follow-up period in the GenSalt study. Panel A is for the FPG change (95% CI) and Panel B is for the accumulative T2D incidence (95% CI) according to the quartiles of the number of risk alleles in the GenSalt participants. FPG, fasting plasma glucose; T2D, type 2 diabetes.

The replicated SNPs showed cumulative effects on FPG change and T2D incidence over a follow-up of 7.5 years among the GenSalt participants. A total of 1,634 participants (86.9%) were examined in the follow-up studies and 126 participants developed T2D during the follow-up. Subjects with more risk alleles had a greater FPG increase and T2D incidence (Figure 1). On average, each quartile increase in the number of risk alleles was associated with a 0.06 mmol/l greater increase in FPG (*P* = 0.03 for trend across quartiles) and 19% higher odds of developing T2D (*P* = 0.058 for trend across quartiles) during the follow-up.

## Discussion

In this study we replicated the association of several genomic loci, which were previously predominately reported in GWAS of European populations, with T2D among a large Han Chinese population. We also observed that most of the replicated variants had comparable effects between these two different populations. In addition, some of the variants were associated with quantitative glycemic traits, highlighting their potential effects on β-cell dysfunction and insulin resistance. More notably, we observed that the cumulative effects of replicated variants predicted FPG increase and T2D development among the Chinese population over time.

The locus including *CDKN2A/2B*-rs10811661 showed the most significant association with T2D in the current study of Han Chinese. This locus was initially identified by several GWAS of European descent [17]–[19]. There was a significant difference in the risk allele frequency between Chinese (57.7%) and European populations (80.4%) based on HapMap data. However, the OR for each risk allele T of the lead-SNP rs10811661 (1.21) observed in Han Chinese was very close to that in European populations (ranging from 1.19 to 1.20) [17]–[19]. Consistent findings across ethnicities may highlight the important role of this locus in the pathogenesis of T2D. In the Meta-Analyses of Glucose- and Insulin-related traits Consortium (MAGIC), the risk allele of rs10811661 was associated with higher fasting glucose among individuals of European ancestry (β = 0.017, *P* = 2.72×10^−5^) [20]. Although this association with fasting glucose was not replicated in either our study or a previous study of Han Chinese [5], we did observe that this SNP was associated with 2-h glucose during OGTT and the insulinogenic index. These findings suggest that this locus may be involved in insufficient insulin secretion of β-cells in response to glucose challenge.

A previous GWAS of Han Chinese failed to replicate the association of *CDC123*-rs12779790 with T2D, but identified an adjacent SNP rs10906115 (about 13 kb away from rs12779790, r^2^ = 0.196 based on the HapMap CHB data) associated with T2D [21]. Our study replicated the association of *CDC123*-rs12779790 with T2D in Han Chinese for the first time (*P* = 0.002), although the AGEN meta-analysis has replicated this locus among East Asians (*P* = 0.01). These findings indicate that this locus may have at least two independent signals regarding the association with T2D. The associations with fasting insulin and HOMA-IR suggest that this locus may play a role in insulin resistance in the pathogenesis of T2D.

Our study not only replicated the associations of *CDKAL1*-rs7756992 and *HHEX*-rs1111875 with T2D in Han Chinese, but also confirmed their associations with β-cell function. The risk alleles of these two SNPs were significantly associated with a lower insulinogenic index measured through the OGTT. These findings were consistent with those observed in Europeans [22], [23]. In addition, we observed that *MTNR1B*-rs10830963 was associated with T2D and FPG in the Han Chinese of the DMS study, which was also consistent with the finding in Europeans [20].

*TCF7L2*, the susceptibility gene with the largest effect on T2D discovered to date, was identified pre-GWAS in 2006 [24], with rapid replication by subsequent GWAS among European populations [17], [18], [20], [25]–[30]. The *TCF7L2* gene has been linked to β-cell function [31], and SNP *TCF7L2*-rs7903146 has allelic-specific enhancer effects on the *TCF7L2* gene, which might explain its association with T2D [32]. Although it exhibited a strong effect on T2D among Europeans, the association of *TCF7L2*-rs7903146 with T2D in Han Chinese has not been well replicated previously. The most likely reason is the relatively low risk allele frequency of rs7903146 in Han Chinese compared to Europeans (2.6% vs. 27.9%). In this large replication study, we did replicate the association of this SNP with T2D and also observed its association with 2-h glucose level during the OGTT.

Variants in *KCNQ1* were first identified in Japanese and replicated in European and South Asian populations [33]–[35]. Our study further confirmed its association with T2D in the Han Chinese population. A meta-analysis of the *FTO* gene in East Asians (17,255 case and 19,703 control subjects) has shown variants of *FTO* associated with both obesity and T2D [36]. After adjusting for BMI, *FTO*-rs9939609 showed borderline significance (*P* = 0.07) in the DMS sample, with the direction of association consistent with the AGEN meta-analysis. Although BMI was not adjusted in the AGEN analysis, the combined effect from DMS and AGEN (OR [95% CI] = 1.13 [1.08, 1.19]) was close to that from the aforementioned *FTO* meta-analysis (OR [95% CI] = 1.10 [1.03, 1.17]), in which BMI was adjusted in included studies.

Longitudinal replication is necessary to confirm the role of the GWAS-identified variants in the development of a disease and to assess the predictive value of the genetic markers on disease risk. Very few longitudinal studies have examined the effects of GWAS-T2D loci on the incidence of T2D among Han Chinese [7], [37]. Our study provided further evidence for the cumulative effect of these replicated loci, most of which have small to moderate effects, on the increase in FPG and the risk of developing T2D.

To the best of our knowledge, this is the largest replication study of GWAS-T2D loci among Han Chinese. Genetic homogeneity of study participants further improved the study power. Both cross-sectional and longitudinal replication analyses were implemented in the study. In addition, a series of glucose metabolism measurements was conducted and analyzed to explore possible mechanisms of replicated genetic factors. However, our study has some limitations. First, only the reported lead SNP from each locus was tested. This approach may fail to replicate some loci in which the lead SNPs had different linkage disequilibrium with causal variants between Caucasians and Han Chinese. Second, many associations were only significant at α = 0.05 and could not tolerate correction for multiple testing. However, it should be noted that this study is a replication of previous GWAS findings with high prior probability, so a stringent threshold may not be necessary for statistical significance.

In summary, our large and comprehensive analyses replicated the associations of several GWAS-T2D loci, established in European populations, with T2D and a variety of quantitative glycemic traits in Han Chinese populations. The cross-ethnicity replication of these T2D-related loci further highlights their importance in the genetic basis of this disease. Future studies on fine mapping causal variants within these loci are necessary to understand the mechanism underlying these replicated associations.

## Supporting Information

File S1 **Information of genotyped SNPs and associations of all genotyped SNPs with type-2 diabetes and quantitative glycemic traits.**
(DOCX)
