# Supplementary material for: Cross-Sectional and Longitudinal Replication Analyses of Genome-Wide Association Loci of Type 2 Diabetes in Han Chinese
Source: PLoS One. 2014 Mar 17;9(3):e91790. doi: 10.1371/journal.pone.0091790 (PMC3956742; doi:10.1371/journal.pone.0091790)
Supplement: File S1 — Information of genotyped SNPs and associations of all genotyped SNPs with type-2 diabetes and quantitative glycemic traits. (DOCX) [file pone.0091790.s001.docx]

| **Table S1. Information of genotyped SNPs in this study** | | | | | | | |
| --- | --- | --- | --- | --- | --- | --- | --- |
| SNP | Chr | Position  (Build 37) | Nearby gene | Alleles^a^ | MAF | MAF  (CHB/CEU)^b^ | HW-*P* |
| rs10923931 | 1 | 120517959 | *NOTCH2* | G:**T** | 0.040 | 0.024/0.094 | 0.96 |
| rs780094 | 2 | 27741237 | *GCKR* | A:**G** | 0.476 | 0.387/0.607 | 0.36 |
| rs7578597 | 2 | 43732823 | *THADA* | **T**:C | 0.007 | 0.006/0.125 | 0.44 |
| rs243021 | 2 | 60584819 | *BCL11A^c^* | **T**:C | 0.320 | 0.381/0.522 | 0.76 |
| rs1801282 | 3 | 12393125 | *PPARG* | **C**:G | 0.065 | 0.048/0.098 | 1.00 |
| rs4607103 | 3 | 64711904 | *ADAMTS9^c^* | **C**:T | 0.377 | 0.423/0.188 | 0.05 |
| rs10010131 | 4 | 6292915 | *WFS1* | **G**:A | 0.046 | 0.036/0.326 | 0.43 |
| rs4457053 | 5 | 76424949 | *ZBED3^c^* | A:**G** | 0.050 | 0.036/0.259 | 0.56 |
| rs7756992 | 6 | 20679709 | *CDKAL1* | **G**:A | 0.477 | 0.524/0.728 | 0.05 |
| rs864745 | 7 | 28180556 | *JAZF1* | **A**:G | 0.239 | 0.238/0.513 | 1.00 |
| rs972283 | 7 | 130466854 | *KLF14^c^* | **G**:A | 0.279 | 0.333/0.450 | 0.19 |
| rs896854 | 8 | 95960511 | *TP53INP1* | G:**A** | 0.341 | 0.271/0.436 | 0.02 |
| rs10811661 | 9 | 22134094 | *CDKN2A/B^c^* | **T**:C | 0.476 | 0.423/0.196 | 0.52 |
| rs13292136 | 9 | 81952128 | *CHCHD9^c^* | **C**:T | 0.094 | 0.089/0.067 | 0.62 |
| rs12779790 | 10 | 12328010 | *CDC123^c^* | A:**G** | 0.165 | 0.133/0.229 | 0.79 |
| rs1111875 | 10 | 94462882 | *HHEX^c^* | A:**G** | 0.284 | 0.315/0.585 | 1.00 |
| rs7903146 | 10 | 114758349 | *TCF7L2* | C:**T** | 0.039 | 0.024/0.281 | 0.32 |
| rs2237895 | 11 | 2857194 | *KCNQ1* | A:**C** | 0.320 | 0.300/0.358^d^ | 0.37 |
| rs1552224 | 11 | 72433098 | *ARAP1* | **T**:G | 0.090 | 0.089/0.129 | 0.37 |
| rs10830963 | 11 | 92708710 | *MTNR1B* | C:**G** | 0.413 | 0.417/0.300 | 0.36 |
| rs7961581 | 12 | 71663102 | *TSPAN8^c^* | T:**C** | 0.205 | 0.196/0.254 | 0.15 |
| rs7957197 | 12 | 121460686 | *OASL* | **T**:A | 0.003 | 0.000/0.150 | 1.00 |
| rs11634397 | 15 | 80432222 | *ZFAND6^c^* | A:**G** | 0.099 | 0.071/0.643 | 0.37 |
| rs8042680 | 15 | 91521337 | *PRC1* | **A**:C | 0.019 | 0.006/0.741 | 0.98 |
| rs9939609 | 16 | 53820527 | *FTO* | T:**A** | 0.113 | 0.149/0.460 | 0.43 |
| ^a^ Major allele: minor allele; previously reported risk alleles are shown in bold. ^b^ The allele frequencies of the minor allele in this study in HapMap CHB and CEU populations. ^c^ The nearest gene is provided if a SNP is intergenic. ^d^ Allele frequencies are based on the 1000 Genome project. HW-P, Hardy-Weinberg equilibrium test P value; MAF, minor allele frequency. | | | | | | | |

| **Table S2. Associations of all genotyped loci with T2D in the DMS, AGEN, and combined DMS+AGEN studies** | | | | | | | | | | | | | | | | | |
| --- | --- | --- | --- | --- | --- | --- | --- | --- | --- | --- | --- | --- | --- | --- | --- | --- | --- |
|  | SNP | Nearby gene | Effect  alleles^a^ | DMS | |  | DMS  (with adjustment for BMI) | |  | AGEN^b^ | |  | DMS + AGEN | | |  | Reported  OR^d^ |
|  |  |  |  | OR (95% CI) | *P*-value |  | OR (95% CI) | *P*-value |  | OR (95% CI) | *P*-value |  | OR (95% CI) | *P*-value | N^c^ |  |  |
| 1 | rs10923931 | *NOTCH2* | T | 0.92 (0.79-1.07) | 2.30×10^-1^ |  | 0.91 (0.77-1.07) | 2.65×10^-1^ |  | 1.05 (0.92-1.20) | 4.52×10^-1^ |  | 0.99 (0.90-1.10) | 8.91×10^-1^ | 28,731 |  | 1.13 |
| 2 | rs780094 | *GCKR* | G | 1.05 (1.00-1.12) | 7.31×10^-2^ |  | 1.06 (0.99-1.13) | 7.89×10^-2^ |  | 1.05 (1.01-1.10) | 2.04×10^-2^ |  | 1.05 (1.02-1.09) | **4.52×10^-3^** | 28,667 |  | 1.06 |
| 3 | rs7578597 | *THADA* | T | 1.02 (0.73-1.43) | 8.94×10^-1^ |  | 0.99 (0.67-1.44) | 9.38×10^-1^ |  | 0.93 (0.62-1.40) | 7.37×10^-1^ |  | 0.96 (0.73-1.27) | 7.68×10^-1^ | 15,841 |  | 1.15 |
| 4 | rs243021 | *BCL11A^e^* | T | 1.04 (0.98-1.11) | 1.99×10^-1^ |  | 1.02 (0.96-1.10) | 4.91×10^-1^ |  | 1.05 (1.00-1.10) | 4.02×10^-2^ |  | 1.04 (1.00-1.08) | **4.12×10^-2^** | 28,482 |  | 1.08 |
| 5 | rs1801282 | *PPARG* | C | 1.06 (0.94-1.19) | 3.45×10^-1^ |  | 1.10 (0.96-1.25) | 1.66×10^-1^ |  | 1.13 (1.01-1.28) | 3.92×10^-2^ |  | 1.12 (1.02-1.22) | **1.51×10^-2^** | 26,654 |  | 1.14 |
| 6 | rs4607103 | *ADAMTS9^e^* | C | 1.03 (0.97-1.09) | 3.37×10^-1^ |  | 1.05 (0.99-1.13) | 1.28×10^-1^ |  | 0.99 (0.95-1.04) | 6.74×10^-1^ |  | 1.01 (0.97-1.05) | 6.20×10^-1^ | 28,557 |  | 1.09 |
| 7 | rs10010131 | *WFS1* | G | 1.19 (1.03-1.37) | **1.55×10^-2^** |  | 1.21 (1.04-1.41) | **1.67×10^-2^** |  | 1.00 (0.91-1.10) | 9.92×10^-1^ |  | 1.05 (0.97-1.14) | 2.14×10^-1^ | 28,727 |  | 1.11 |
| 8 | rs4457053 | *ZBED3^e^* | G | 0.96 (0.84-1.09) | 6.05×10^-1^ |  | 0.94 (0.82-1.09) | 4.32×10^-1^ |  | 1.00 (0.85-1.18) | 9.77×10^-1^ |  | 0.97 (0.87-1.08) | 5.59×10^-1^ | 15,630 |  | 1.08 |
| 9 | rs7756992 | *CDKAL1* | G | 1.12 (1.06-1.19) | **7.30×10^-5^** |  | 1.16 (1.08-1.23) | **1.02×10^-5^** |  | - | - |  | - | - | - |  | 1.20 |
| 10 | rs864745 | *JAZF1* | A | 1.02 (0.95-1.09) | 6.25×10^-1^ |  | 1.04 (0.96-1.12) | 3.66×10^-1^ |  | 1.06 (1.00-1.12) | 3.50×10^-2^ |  | 1.05 (1.00-1.10) | **3.13×10^-2^** | 28,457 |  | 1.10 |
| 11 | rs972283 | *KLF14^e^* | G | 0.99 (0.93-1.05) | 8.18×10^-1^ |  | 0.99 (0.92-1.07) | 7.43×10^-1^ |  | 0.99 (0.93-1.06) | 8.52×10^-1^ |  | 0.99 (0.94-1.04) | 6.57×10^-1^ | 20,599 |  | 1.07 |
| 12 | rs896854 | *TP53INP1* | A | 1.03 (0.97-1.10) | 2.78×10^-1^ |  | 1.02 (0.96-1.10) | 4.88×10^-1^ |  | 1.07 (1.02-1.12) | 9.05×10^-3^ |  | 1.06 (1.02-1.10) | **6.30×10^-3^** | 28,485 |  | 1.06 |
| 13 | rs10811661 | *CDKN2A/B^e^* | T | 1.17 (1.10-1.24) | **1.45×10^-7^** |  | 1.21 (1.13-1.29) | **1.11×10^-8^** |  | 1.21 (1.14-1.28) | 6.04×10^-11^ |  | 1.21 (1.16-1.26) | **6.87×10^-18^** | 22,665 |  | 1.19 |
| 14 | rs13292136 | *CHCHD9^e^* | C | 0.97 (0.88-1.07) | 5.94×10^-1^ |  | 0.96 (0.86-1.07) | 5.24×10^-1^ |  | 0.99 (0.92-1.07) | 8.84×10^-1^ |  | 0.98 (0.92-1.04) | 5.64×10^-1^ | 28,655 |  | 1.11 |
| 15 | rs12779790 | *CDC123^e^* | G | 1.09 (1.01-1.18) | **2.17×10^-2^** |  | 1.14 (1.05-1.24) | **2.27×10^-3^** |  | 1.12 (1.02-1.23) | 1.31×10^-2^ |  | 1.13 (1.06-1.20) | **1.16×10^-4^** | 18,674 |  | 1.11 |
| 16 | rs1111875 | *HHEX^e^* | G | 1.12 (1.05-1.19) | **5.75×10^-4^** |  | 1.13 (1.05-1.21) | **8.05×10^-4^** |  | 1.11 (1.06-1.18) | 8.74×10^-5^ |  | 1.12 (1.07-1,17) | **4.09×10^-7^** | 24,644 |  | 1.13 |
| 17 | rs7903146 | *TCF7L2* | T | 1.31 (1.14-1.51) | **1.71×10^-4^** |  | 1.34 (1.15-1.57) | **1.97×10^-4^** |  | 1.16 (1.02-1.31) | 2.50×10^-2^ |  | 1.23 (1.11-1.36) | **3.41×10^-5^** | 28,652 |  | 1.40 |
| 18 | rs2237895 | *KCNQ1* | C | 1.20 (1.12-1.27) | **5.63×10^-8^** |  | 1.22 (1.13-1.31) | **5.45×10^-8^** |  | - | - |  | - | **-** | - |  | 1.29 |
| 19 | rs1552224 | *ARAP1* | T | 1.07 (0.96-1.18) | 2.06×10^-1^ |  | 1.06 (0.95-1.19) | 3.14×10^-1^ |  | 1.16 (1.06-1.27) | 1.58×10^-3^ |  | 1.12 (1.04-1.20) | **1.69×10^-3^** | 28,654 |  | 1.14 |
| 20 | rs10830963 | *MTNR1B* | G | 1.07 (1.00-1.13) | **3.55×10^-2^** |  | 1.08 (1.01-1.15) | **2.84×10^-2^** |  | 0.99 (0.93-1.06) | 8.64×10^-1^ |  | 1.03 (0.99-1.08) | 1.80×10^-1^ | 18,504 |  | 1.09 |
| 21 | rs7961581 | *TSPAN8^e^* | C | 1.07 (1.00-1.15) | 6.83×10^-2^ |  | 1.05 (0.97-1.14) | 2.28×10^-1^ |  | 1.01 (0.95-1.06) | 8.49×10^-1^ |  | 1.02 (0.98-1.07) | 3.28×10^-1^ | 28,577 |  | 1.09 |
| 22 | rs7957197 | *OASL* | T | 1.05 (0.64-1.73) | 8.49×10^-1^ |  | 0.99 (0.56-1.76) | 9.67×10^-1^ |  | - | - |  | - | - | - |  | 1.07 |
| 23 | rs11634397 | *ZFAND6^e^* | G | 1.03 (0.94-1.14) | 5.06×10^-1^ |  | 1.02 (0.92-1.14) | 6.75×10^-1^ |  | 0.99 (0.90-1.09) | 8.29×10^-1^ |  | 1.00 (0.94-1.08) | 8.97×10^-1^ | 22,638 |  | 1.06 |
| 24 | rs8042680 | *PRC1* | A | 1.08 (0.87-1.33) | 4.97×10^-1^ |  | 1.12 (0.89-1.42) | 3.30×10^-1^ |  | 1.64 (1.16-2.32) | 4.92×10^-3^ |  | 1.27 (1.04-1.54) | **1.73×10^-2^** | 15,888 |  | 1.07 |
| 25 | rs9939609 | *FTO* | A | 1.17 (1.07-1.28) | **4.84×10^-4^** |  | 1.09 (0.99-1.21) | 7.23×10^-2^ |  | 1.15 (1.08-1.22) | 6.85×10^-6^ |  | 1.13 (1.08-1.19) | **1.91×10^-6^** | 28,678 |  | 1.34 |
| ^a^ Previously reported risk alleles. ^b^ Data of ORs and P values are from the T2D meta-analysis of the AGEN consortium. ^c^ Total number of individuals were used in the combined DMS and AGEN analysis. ^d^ Previously reported effects mainly among Europeans. ^e^ The nearest gene is provided if a SNP is intergenic. AGEN, the Asian Genetic Epidemiology Network; DMS, the China National Diabetes and Metabolic Disorders study; OR, odds ratio; SNP, single nucleotide polymorphism. | | | | | | | | | | | | | | | | | |

| **Table S3. Associations of all genotyped loci with quantitative glycemic traits in controls of the DMS case-control sample** | | | | | | | | | | | | | | | | | | | | |
| --- | --- | --- | --- | --- | --- | --- | --- | --- | --- | --- | --- | --- | --- | --- | --- | --- | --- | --- | --- | --- |
|  | SNP | Nearby  gene | Effect  allele^a^ | Fasting glucose  (mmol) | |  | OGTT 2-h glucose  (mmol/l) | |  | Fasting insulin  (pmol/l) | |  | HOMA_IR | |  | HOMA_B | |  | Insulinogenic index | |
|  |  |  |  | β (SE) | *P*-value |  | β (SE) | *P*-value |  | β (SE) | *P*-value |  | β (SE) | *P*-value |  | β (SE) | *P*-value |  | β (SE) | *P*-value |
| 1 | rs10923931 | *NOTCH2* | T | 0 (0.03) | 8.99×10^-1^ |  | -0.05 (0.06) | 3.94×10^-1^ |  | 0.01 (0.03) | 8.41×10^-1^ |  | 0 (0.03) | 8.58×10^-1^ |  | 0.04 (0.04) | 2.58×10^-1^ |  | -0.03 (0.06) | 5.90×10^-1^ |
| 2 | rs780094 | *GCKR* | G | 0.02 (0.01) | 6.87×10^-2^ |  | 0.03 (0.02) | 1.76×10^-1^ |  | 0.01 (0.01) | 4.25×10^-1^ |  | 0.01 (0.01) | 2.11×10^-1^ |  | -0.01 (0.01) | 5.23×10^-1^ |  | 0.05 (0.02) | **4.67×10^-2^** |
| 3 | rs7578597 | *THADA* | T | 0.01 (0.06) | 8.16×10^-1^ |  | -0.21 (0.13) | 1.02×10^-1^ |  | -0.1 (0.05) | 7.36×10^-2^ |  | -0.09 (0.06) | 1.16×10^-1^ |  | -0.12 (0.08) | 1.12×10^-1^ |  | 0.08 (0.13) | 5.30×10^-1^ |
| 4 | rs243021 | *BCL11A^b^* | T | 0.02 (0.01) | 5.49×10^-2^ |  | -0.03 (0.02) | 2.43×10^-1^ |  | 0.02 (0.01) | 8.40×10^-2^ |  | 0.02 (0.01) | **3.36×10^-2^** |  | 0 (0.01) | 9.11×10^-1^ |  | 0.02 (0.03) | 4.12×10^-1^ |
| 5 | rs1801282 | *PPARG* | C | 0.01 (0.02) | 6.55×10^-1^ |  | -0.01 (0.05) | 8.61×10^-1^ |  | 0.02 (0.02) | 3.61×10^-1^ |  | 0.02 (0.02) | 4.20×10^-1^ |  | 0.02 (0.03) | 5.01×10^-1^ |  | 0.08 (0.05) | 1.02×10^-1^ |
| 6 | rs4607103 | *ADAMTS9^b^* | C | 0.01 (0.01) | 2.51×10^-1^ |  | 0.02 (0.02) | 4.54×10^-1^ |  | 0 (0.01) | 6.76×10^-1^ |  | 0.01 (0.01) | 5.59×10^-1^ |  | 0 (0.01) | 9.46×10^-1^ |  | -0.07 (0.02) | **5.33×10^-3^** |
| 7 | rs10010131 | *WFS1* | G | 0.03 (0.03) | 2.31×10^-1^ |  | 0 (0.05) | 9.64×10^-1^ |  | 0.01 (0.02) | 6.07×10^-1^ |  | 0.02 (0.02) | 4.59×10^-1^ |  | 0 (0.03) | 9.48×10^-1^ |  | -0.02 (0.06) | 7.52×10^-1^ |
| 8 | rs4457053 | *ZBED3^b^* | G | 0.01 (0.02) | 7.72×10^-1^ |  | 0.06 (0.05) | 2.54×10^-1^ |  | 0.01 (0.02) | 7.75×10^-1^ |  | 0.01 (0.02) | 7.47×10^-1^ |  | -0.01 (0.03) | 7.94×10^-1^ |  | 0.03 (0.05) | 5.85×10^-1^ |
| 9 | rs7756992 | *CDKAL1* | G | 0.02 (0.01) | 8.90×10^-2^ |  | 0.04 (0.02) | 5.50×10^-2^ |  | -0.01 (0.01) | 2.13×10^-1^ |  | -0.01 (0.01) | 3.65×10^-1^ |  | -0.02 (0.01) | 1.18×10^-1^ |  | -0.07 (0.02) | **6.21×10^-3^** |
| 10 | rs864745 | *JAZF1* | A | 0 (0.01) | 7.69×10^-1^ |  | 0 (0.03) | 9.48×10^-1^ |  | 0 (0.01) | 7.14×10^-1^ |  | 0 (0.01) | 8.01×10^-1^ |  | 0.01 (0.02) | 3.73×10^-1^ |  | -0.02 (0.03) | 4.98×10^-1^ |
| 11 | rs972283 | *KLF14^b^* | G | -0.01 (0.01) | 3.37×10^-1^ |  | 0 (0.03) | 9.34×10^-1^ |  | -0.01 (0.01) | 5.39×10^-1^ |  | -0.01 (0.01) | 3.75×10^-1^ |  | 0.01 (0.02) | 6.28×10^-1^ |  | 0 (0.03) | 9.99×10^-1^ |
| 12 | rs896854 | *TP53INP1* | A | 0.01 (0.01) | 2.00×10^-1^ |  | -0.01 (0.02) | 6.49×10^-1^ |  | -0.03 (0.01) | **1.45×10^-2^** |  | -0.02 (0.01) | **2.65×10^-2^** |  | -0.03 (0.01) | **4.14×10^-2^** |  | -0.03 (0.03) | 3.23×10^-1^ |
| 13 | rs10811661 | *CDKN2A/B^b^* | T | 0.01 (0.01) | 4.89×10^-1^ |  | 0.06 (0.02) | **9.11×10^-3^** |  | 0 (0.01) | 9.60×10^-1^ |  | 0 (0.01) | 7.75×10^-1^ |  | -0.01 (0.01) | 5.29×10^-1^ |  | -0.05 (0.02) | **2.71×10^-2^** |
| 14 | rs13292136 | *CHCHD9^b^* | C | -0.02 (0.02) | 4.20×10^-1^ |  | -0.08 (0.04) | **3.29×10^-2^** |  | -0.02 (0.02) | 3.22×10^-1^ |  | -0.02 (0.02) | 2.80×10^-1^ |  | -0.01 (0.02) | 7.81×10^-1^ |  | -0.04 (0.04) | 3.25×10^-1^ |
| 15 | rs12779790 | *CDC123^b^* | G | 0.01 (0.01) | 6.03×10^-1^ |  | 0.02 (0.03) | 4.17×10^-1^ |  | 0.03 (0.01) | **8.58×10^-3^** |  | 0.04 (0.01) | **9.39×10^-3^** |  | 0.02 (0.02) | 1.78×10^-1^ |  | 0 (0.03) | 9.94×10^-1^ |
| 16 | rs1111875 | *HHEX^b^* | G | -0.02 (0.01) | 1.18×10^-1^ |  | -0.01 (0.03) | 7.85×10^-1^ |  | -0.01 (0.01) | 2.93×10^-1^ |  | -0.02 (0.01) | 1.67×10^-1^ |  | 0.01 (0.02) | 7.13×10^-1^ |  | -0.09 (0.03) | **6.73×10^-4^** |
| 17 | rs7903146 | *TCF7L2* | T | 0.03 (0.03) | 3.39×10^-1^ |  | 0.17 (0.06) | **4.90×10^-3^** |  | 0.03 (0.03) | 2.55×10^-1^ |  | 0.03 (0.03) | 2.36×10^-1^ |  | 0.02 (0.04) | 4.81×10^-1^ |  | -0.01 (0.06) | 8.66×10^-1^ |
| 18 | rs2237895 | *KCNQ1* | C | 0 (0.01) | 8.26×10^-1^ |  | -0.01 (0.03) | 6.92×10^-1^ |  | 0 (0.01) | 6.50×10^-1^ |  | 0 (0.01) | 6.90×10^-1^ |  | -0.01 (0.01) | 5.76×10^-1^ |  | -0.03 (0.03) | 2.38×10^-1^ |
| 19 | rs1552224 | *ARAP1* | T | 0.02 (0.02) | 2.14×10^-1^ |  | 0.02 (0.04) | 6.12×10^-1^ |  | -0.01 (0.02) | 7.28×10^-1^ |  | 0 (0.02) | 9.92×10^-1^ |  | -0.03 (0.02) | 2.20×10^-1^ |  | -0.06 (0.04) | 1.74×10^-1^ |
| 20 | rs10830963 | *MTNR1B* | G | 0.03 (0.01) | **5.50×10^-3^** |  | 0 (0.02) | 8.56×10^-1^ |  | 0 (0.01) | 8.33×10^-1^ |  | 0.01 (0.01) | 4.34×10^-1^ |  | -0.02 (0.01) | 1.63×10^-1^ |  | -0.02 (0.02) | 4.31×10^-1^ |
| 21 | rs7961581 | *TSPAN8^b^* | C | 0.01 (0.01) | 3.69×10^-1^ |  | 0.03 (0.03) | 3.40×10^-1^ |  | 0 (0.01) | 8.01×10^-1^ |  | 0 (0.01) | 9.36×10^-1^ |  | -0.02 (0.02) | 2.72×10^-1^ |  | -0.04 (0.03) | 1.62×10^-1^ |
| 22 | rs7957197 | *OASL* | T | -0.18 (0.09) | 5.41×10^-2^ |  | 0.15 (0.2) | 4.60×10^-1^ |  | -0.07 (0.09) | 4.43×10^-1^ |  | -0.11 (0.09) | 2.46×10^-1^ |  | 0.07 (0.12) | 5.58×10^-1^ |  | 0.18 (0.21) | 3.87×10^-1^ |
| 23 | rs11634397 | *ZFAND6^b^* | G | -0.03 (0.02) | 1.23×10^-1^ |  | -0.05 (0.04) | 2.08×10^-1^ |  | 0 (0.02) | 7.80×10^-1^ |  | 0 (0.02) | 9.74×10^-1^ |  | 0.03 (0.02) | 1.46×10^-1^ |  | -0.11 (0.04) | **4.67×10^-3^** |
| 24 | rs8042680 | *PRC1* | A | 0.01 (0.04) | 8.99×10^-1^ |  | -0.03 (0.08) | 7.41×10^-1^ |  | -0.02 (0.04) | 6.45×10^-1^ |  | -0.02 (0.04) | 6.72×10^-1^ |  | -0.03 (0.05) | 5.52×10^-1^ |  | 0 (0.08) | 9.98×10^-1^ |
| 25 | rs9939609 | *FTO* | A | -0.01 (0.02) | 7.52×10^-1^ |  | 0 (0.04) | 8.97×10^-1^ |  | -0.04 (0.02) | **2.26×10^-2^** |  | -0.04 (0.02) | **1.82×10^-2^** |  | -0.02 (0.02) | 3.29×10^-1^ |  | 0.01 (0.04) | 7.27×10^-1^ |
| ^a^ Previously reported risk alleles. ^b^ The nearest gene is provided if a SNP is intergenic. HOMA-B, homoeostasis model assessment of beta-cell function; HOMA-IR, homoeostasis model assessment of insulin resistance; OGTT, oral glucose tolerance test; SE, standard error; SNP, single nucleotide polymorphism. | | | | | | | | | | | | | | | | | | | | |
